# Isolated Silymarin Flavonoids Increase Systemic and Hepatic Bilirubin Concentrations and Lower Lipoperoxidation in Mice

**DOI:** 10.1155/2019/6026902

**Published:** 2019-02-12

**Authors:** Jakub Šuk, Jana Jašprová, David Biedermann, Lucie Petrásková, Kateřina Valentová, Vladimír Křen, Lucie Muchová, Libor Vítek

**Affiliations:** ^1^Institute of Medical Biochemistry and Laboratory Diagnostics, 1st Faculty of Medicine, Charles University, Kateřinská 32, Praha 2 12000, Czech Republic; ^2^Laboratory of Biotransformation, Institute of Microbiology of the Czech Academy of Sciences, Vídeňská 1082, Praha 4 14000, Czech Republic; ^3^4th Department of Internal Medicine, 1st Faculty of Medicine, Charles University, Kateřinská 32, Praha 2 12000, Czech Republic

## Abstract

Bilirubin is considered to be one of the most potent endogenous antioxidants in humans. Its serum concentrations are predominantly affected by the activity of hepatic bilirubin UDP-glucuronosyl transferase (UGT1A1). Our objective was to analyze the potential bilirubin-modulating effects of natural polyphenols from milk thistle (*Silybum marianum*), a hepatoprotective herb. Human hepatoblastoma HepG2 cells were exposed to major polyphenolic compounds isolated from milk thistle. Based on *in vitro* studies, 2,3-dehydrosilybins A and B were selected as the most efficient compounds and applied either intraperitoneally or orally for seven days to C57BL/6 mice. After, *UGT1A1* mRNA expression, serum, intrahepatic bilirubin concentrations, and lipoperoxidation in the liver tissue were analyzed. All natural polyphenols used increased intracellular concentration of bilirubin in HepG2 cells to a similar extent as atazanavir, a known bilirubinemia-enhancing agent. Intraperitoneal application of 2,3-dehydrosilybins A and B (the most efficient flavonoids from *in vitro* studies) to mice (50 mg/kg) led to a significant downregulation of *UGT1A1* mRNA expression (46 ± 3% of controls, *p* < 0.005) in the liver and also to a significant increase of the intracellular bilirubin concentration (0.98 ± 0.03*vs.*1.21 ± 0.02 nmol/mg, *p* < 0.05). Simultaneously, a significant decrease of lipoperoxidation (61 ± 2% of controls, *p* < 0.005) was detected in the liver tissue of treated animals, and similar results were also observed after oral treatment. Importantly, both application routes also led to a significant elevation of serum bilirubin concentrations (125 ± 3% and 160 ± 22% of the controls after intraperitoneal and oral administration, respectively, *p* < 0.005 in both cases). In conclusion, polyphenolic compounds contained in silymarin, in particular 2,3-dehydrosilybins A and B, affect hepatic and serum bilirubin concentrations, as well as lipoperoxidation in the liver. This phenomenon might contribute to the hepatoprotective effects of silymarin.

## 1. Introduction

Bilirubin, the end product of heme catabolism in the systemic circulation, is a potent antioxidant substance [1]. Despite the fact that for decades bilirubin has been considered a toxic catabolic waste product and an ominous sign of liver dysfunction, its role as a powerful protective molecule has increasingly been recognized [2]. *In vitro* and *in vivo* studies have shown that bilirubin may suppress the oxidation of lipids [1] and has anti-inflammatory [3], antiproliferative [4], antigenotoxic [5], antimutagenic [6], or even anti-aging properties [7]. Interestingly, bilirubin has been reported as a potent peroxisome proliferator-activated receptor-*α* (PPAR*α*) agonist, thus acting as a real endocrine molecule, with all potential clinical consequences [8]. The clinical evidence is even more important. Bilirubin, when only mildly elevated, has been demonstrated to protect from a wide array of oxidative stress-related diseases, including cardiovascular diseases, certain cancers, and autoimmune or neurodegenerative diseases [2, 9]. In fact, substantial protective effects of mild unconjugated hyperbilirubinemia, as seen in subjects with Gilbert syndrome (benign hyperbilirubinemia), have been reported for atherosclerotic diseases in particular [10].

Bilirubin production is dependent on heme oxygenase (HMOX) activity, but systemic bilirubin concentrations are predominantly affected by hepatic bilirubin UDP-glucuronosyl transferase (UGT1A1), its biotransforming enzyme [11]. Partial inhibition of bilirubin glucuronosylation was proposed as a wise strategy used to induce “iatrogenic” Gilbert syndrome [12]. This indeed was demonstrated as a “side effect” of several drugs used in various indications, typically for atazanavir, whose administration is often associated with mildly elevated concentrations of unconjugated bilirubin. Surprisingly, hyperbilirubinemia induced by atazanavir was reported to decrease markers of oxidative stress [13] and cardiometabolic risk factors [14], as well as endothelial functions [15], but safer approaches are certainly needed.

**Figure 1:**
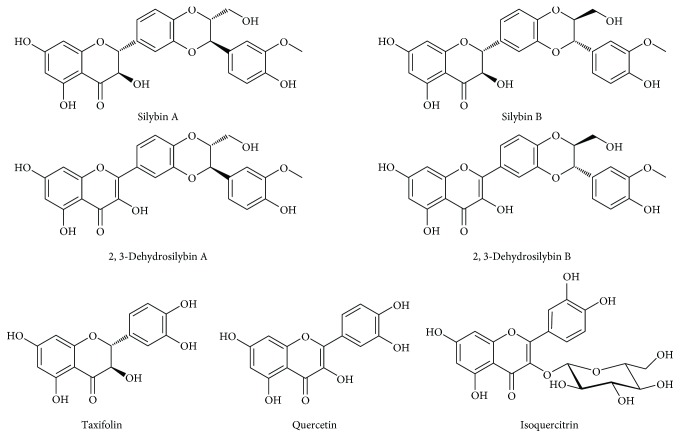
Structures of flavonolignans of the silymarin complex and related flavonols.

Since xenobiotics used in clinical medicine are often associated with potentially severe side effects, natural compounds interfering with the UGT1A1 hepatic biotransformation system seem a better strategy to induce mild hyperbilirubinemia. Based on the scarce data reported in the literature (mainly as the result of investigating potential nutraceuticals/herb-drug interactions), it seems that flavonoids from silymarin might modulate this enzyme. This assumption is based on the fact that many herbal extracts including silymarin, a seed extract of milk thistle (*Silybum marianum* (L.) Gaertn.), are rich in phenolic phytochemicals that are substrates for UGT1A1 or even exhibit UGT1A1-inhibiting activities [16–18]. Indeed, therapy for prostate cancer patients with high doses of silybin (silibinin) has been associated with unconjugated hyperbilirubinemia, which was considered by the authors as an adverse effect of such treatment [19]. Similar findings were also reported in hepatitis C patients receiving silybin therapy [20–23]. Although most of the experimental reports as well as some clinical data suggest its beneficial role, silymarin is generally considered to have a negligible importance clinically [24]. There are many possible reasons: one of them being the poorly defined content of the active ingredients and also the improperly characterized biological properties of individual pure flavonoids in the silymarin complex [25, 26]. Silymarin is a mixture of 5 major flavonolignans (silybins A and B, isosilybin A, silychristin A, and silydianin) plus their precursor taxifolin, as well as other minor polyphenolic compounds (Figure 1) [27]. Among them, the 2,3-dehydroflavonolignans such as 2,3-dehydrosilybins A and B possess potent biological activities [28–32].

Thus, the aim of our study was to investigate the potential bilirubin-modulating effects of natural polyphenols present in milk thistle and related compounds.

## 2. Materials and Methods

### 2.1. Chemicals

Silymarin (containing 13.0% of silybin A, 17.9% silybin B, 14.7% silychristin A, 9.3% silychristin B+silydianin, 8.9% isosilybin A, 6.8% isosilybin B, 3.0% taxifolin, 1.9% 2,3-dehydrosilybin, 0.5% 2,3-dehydrosilychristin, 6.5% of other nonidentified 2,3-dehydroflavonolignans, plus 17.5% of yet other substances (probably polymers, for details of the analysis, see the Supplementary Data and Supplementary Figures 1–3), silybin AB (approximately an equimolar mixture of silybin A and silybin B), quercetin, and rutin (quercetin-3-*O*-*β*-rutinoside)) was all purchased from Sigma-Aldrich (St. Louis, MO, USA); atazanavir was obtained from Santa Cruz (Santa Cruz Biotechnology, Dallas, TX, USA). Silybin A and silybin B were isolated from silymarin (purchased from Liaoning Senrong Pharmaceutical, Panjin, China; batch no. 120501), as described in [33]. 2,3-Dehydrosilybin, 2,3-dehydrosilybin A, and 2,3-dehydrosilybin B were prepared by oxidation of silybin, silybin A, and silybin B, respectively [34]. Taxifolin hydrate (92%) was purchased from Amagro (Prague, CZ), and isoquercitrin (quercetin-3-*O*-glucoside, 97%) was prepared from rutin (Sigma-Aldrich) using thermophilic *α*-L-rhamnosidase from *Aspergillus terreus* heterologously expressed in *Pichia pastoris* [35]. The deconjugation enzymes *β*-glucuronidase and sulfatase from *Helix pomatia* were obtained from Sigma-Aldrich.

### 2.2. Cell Cultures

The HepG2 human hepatoblastoma cell line was used for the *in vitro* studies (ATCC, Manassas, VA, USA). Cells were cultured in MEM Eagle medium, containing 10% of fetal bovine serum, in 75 cm^2^ culture flasks, at 37°C, in a 5% CO_2_ atmosphere. For an estimation of the intracellular bilirubin concentration, the cells were plated in 10 cm Petri dishes and treated with natural polyphenols dissolved in DMSO (vehicle; 0.66%, *v*/*v*) for 24 h. For qPCR measurement, the cells were cultured in 24-well plates and treated with natural flavonoids dissolved in DMSO (vehicle; 0.66%, *v*/*v*) for 4 h.

The cell lines were authenticated at ATCC by STR profiling before distribution and also reauthenticated at the end of the study by an external laboratory (Generi Biotech, Hradec Králové, Czech Republic).

### 2.3. MTT Cytotoxicity Assays

The MTT (3-(4,5-dimethylthiazol-2-yl)-2,5-diphenyltetrazolium bromide) reduction assay in a 96-well format was used for the determination of the cytotoxicity of the compounds tested. HepG2 cells were seeded into a 96-well plate at a density of 1 × 10^5^ cells/well. Following 24 h incubation, the cells were treated for 24 h with natural flavonoids at concentrations of 0.1-200 *μ*M dissolved in DMSO (vehicle; 0.66%, *v*/*v*). MTT assay was measured spectrophotometrically at 540 nm (TECAN, Schoeller Instruments s.r.o., Prague, Czech Republic).

### 2.4. RNA Isolation and Real-Time PCR

Total mRNA was isolated using a 5 Prime PerfectPure RNA Cultured Cell Kit and a 5 Prime PerfectPure RNA Tissue Kit (Eppendorf, Germany); and cDNA was generated using a High Capacity RNA-to-cDNA Master Mix (Life Technologies, Carlsbad, CA, USA) according to the manufacturer's instructions. qPCR for the cell culture was performed using the SYBR™ Select Master Mix (Life Technologies). For each reaction, the final 20 *μ*L volume was comprised of 10 *μ*L of SYBR Green PCR Mix, 2 *μ*L of each primer (Supplementary Table 1), and 6 *μ*L of a 1/5 dilution of the RT products. qPCR for the liver tissue was performed using a TaqMan® Gene Expression Assay Kit (Life Technologies). Threshold cycle (Ct) values were analyzed using the comparative Ct (ΔΔCt) method as recommended by the manufacturer (Applied Biosystems, Foster City, CA, USA). The data were normalized to the expression of hypoxantin phosphoribosyl transferase (*HPRT*) and expressed as the multiplicity change from the control levels.

### 2.5. Serum Markers of Liver Damage

Hepatic enzyme (alanine aminotransferase (ALT), aspartate aminotransferase (AST), and alkaline phosphatase (ALP)) activities were determined on an automatic analyzer (Modular Analyzer, Roche Diagnostics GmbH, Germany) using standard assays.

### 2.6. Determination of Bilirubin

For the determination of bilirubin in HepG2 cells and liver tissue, the biological materials were sonicated on ice and then extracted with methanol/chloroform/hexane 10/5/1 (*v*/*v*/*v*) against PBS buffer (pH 6.2). The lower organic phase was subsequently extracted into 50 *μ*L of carbonate buffer (pH 10) in hexane. The resulting polar droplet was loaded onto a C-8 reverse phase column (Luna 3 *μ*m, 150 × 4.6 mm, Phenomenex, Torrance, CA, USA), and bilirubin was determined using an HPLC Agilent 1200 with a diode array detector (Agilent, Santa Clara, CA, USA) as described earlier [36, 37]. The concentration of bilirubin was calculated in nmol/g of wet tissue or pmol/mg of protein for tissue samples or cell cultures, respectively.

For determination of bilirubin in serum, the LC-MS/MS method was used. Ten *μ*L of serum was mixed with 2.5 *μ*L of internal standard (mesobilirubin, *c* = 20 *μ*mol/L) and deproteinized by 50 *μ*L of 1% BHT in methanol, 50 *μ*L of 0.4% ascorbic acid in methanol, and 200 *μ*L of methanol (pH 11). Five *μ*L of the resulting supernatant was loaded on a Poroshell 120 EC-C18 2.1 *μ*m 3.0 × 50 mm column (Agilent), with a gradient of 1 mM NH_4_F in water (A) and methanol (B) as follows: 60% of B was changed in 5 minutes to 100% of B, with a flow rate of 0.4 mL/min and was kept at 100% of B for 3 minutes, with the gradient of flow rate at 0.5 mL/min. The flow was then maintained at 0.5 mL/min until the end of the gradient program. Phase B was changed back to 60% in 0.1 minutes and was kept at 60% until the end of the program at 10 minutes. The mass spectra were recorded on an Agilent 6470 LC/QQQ (Agilent) LC-MS/MS device with electrospray ionization and multiple reaction monitoring in a positive mode. Conditions of the electrospray ionization source, gas flows, and potentials of the mass spectrometer were manually tuned for high sensitivity and a low signal-to-noise ratio of the analytes: ion spray voltage, 3000 V; source temperature, 250°C; heater gas, 8 L/min; nebulizer, 300 psi; sheath gas temperature, 400°C; sheath gas flow, 12 L/min; and nozzle voltage, 500 V. Within the total scan time of 10 min, *m*/*z* 585.3 → 299.1, 16 eV collision energy (CE) at 5.96 min was monitored for bilirubin and *m*/*z* 589.3 → 301.2, 20 eV CE at 6.084 min for mesobilirubin.

### 2.7. HMOX Activity Assay

Twenty *μ*L of liver sonicate (10%) was incubated for 15 min at 37°C in CO-free septum-sealed vials containing 20 *μ*L of 150 *μ*M heme and 20 *μ*L of 4.5 mM NADPH, as previously described [38]. Blank reaction vials contained potassium phosphate buffer in place of NADPH. The amount of CO generated by the reaction and released into the vial headspace was quantified by gas chromatography (GC) with a reduction gas analyzer (Peak Laboratories, Mountain View, CA, USA). HMOX activity was calculated as pmol CO/h/mg fresh weight and expressed as percentage of the control.

### 2.8. UGT1A1 Inhibition Assay

The UGT activity in the microsomal samples was determined by a UGT-Glo™ assay kit according to the manufacturer's instructions (Promega, Madison, WI, USA). Briefly, microsomes with recombinant UGT1A1 (0.0125 mg/mL) were incubated with 16 mM UDPGA and 20 *μ*M multienzyme substrate with increasing concentrations of 2,3-dehydrosilybins A and B at 37°C for 30 min. Then, 40 *μ*L of reconstituted luciferin detection reagent containing d-cysteine was added, and the luminescent signal was allowed to stabilize for 20 min at room temperature. Luminescence was read on a Synergy II plate reader (BioTek, VT, USA). The data were analyzed using the curve fitting method with GraphPad Prism (GraphPad Software, San Diego, CA).

### 2.9. Malondialdehyde Determination

Malondialdehyde (MDA) in the tissue homogenates was measured according to the method described by Wills [39], with some modifications. A 200 *μ*L aliquot of 10% tissue homogenate was mixed with 2 mL of the thiobarbituric acid-trichloroacetic acid reagent (0.375 and 15%, respectively). The mixture was heated on a water bath at 95°C for 20 min. The solution was then cooled to room temperature. The reaction product (thiobarbituric acid-MDA complex) was extracted by adding 3 mL of *n*-butanol to the above solution. The absorbance of the pink-colored extract in *n*-butanol was measured at 532 nm using a spectrophotometer (Sunrise, Tecan, USA). The amount of MDA was calculated using the molar extinction coefficient of 1.56 × 10^5^ M^−1^ × cm^−1^ and expressed as a percentage of the control.

### 2.10. 2,3-Dehydrosilybin Determination in Sera

2,3-Dehydrosilybin concentrations in serum samples were determined after deconjugation of phase II conjugates (sulfates and glucuronide). The activity of the deconjugation enzymes (*β*-glucuronidase and sulfatase) was checked on the selected glucuronides and sulfates prior to analysis. All the samples were measured in duplicate.

The conjugates in serum samples were deconjugated in acetate buffer (50 mM, pH 5.0) by adding *β*-glucuronidase (440 U) with sulfatase (35 U), incubated for 2 h at 37°C and 600 rpm. The reaction mixture was then freeze-dried, and an internal standard (2,3-dehydrosilychristin, 4.8 *μ*g/mL, 150 *μ*L, in acetonitrile/DMSO 1 : 1) was added. Samples were incubated (1 h, 37°C, 600 rpm) and centrifuged (15 min), and the supernatant was injected into a LC-MS (injection volume 25 *μ*L).

#### 2.10.1. LC-MS Conditions

LC-MS chromatograms and mass spectra were obtained using the Shimadzu Prominence system consisting of a DGU-20A3 mobile phase degasser, two LC-20AD solvent delivery units, a SIL-20AC cooling autosampler, a CTO-10AS column oven with the SPD-M20A diode array detector, plus a LCMS-2020 mass detector with a single quadrupole, equipped with an electrospray ion source (Shimadzu, Kyoto, Japan).

The LC-MS of the serum samples and standard solutions of 2,3-dehydrosilybin were measured on a Chromolith RP-18e (100 × 3 mm) column (Merck) and Chromolith RP-18e (5 × 4.6 mm) precolumn (Merck) (mobile phase: A = 5% acetonitrile, 0.1% HCOOH; B = 80% acetonitrile, 0.1% HCOOH; gradient: 0 min 20% B, 5 min 90% B, 6 min 90% B, and 8-10 min 20% B; flow rate: 0.4 mL/min, 25°C). The concentration of 2,3-dehydrosilybin in the serum was calculated using a calibration curve. The MS parameters were as follows: ESI interface voltage, 4.5 kV; detector voltage, 1.15 kV; nebulizing gas flow, 1.5 mL·min^−1^; drying gas flow, 15 mL·min^−1^; heat block temperature, 200°C; DL temperature, 250°C; negative scan mode, 478.8-481.0 *m*/*z*; and software, LabSolutions ver. 5.75 SP2 (Shimadzu, Kyoto, Japan).

### 2.11. Animal Studies

Female C57BL/6 mice (*n* = at least 6 in each group, 8 w old) obtained from Velaz (Prague, Czech Republic) had access to both water and a standard diet *ad libitum*. An equimolar mixture of dehydrosilybins A and B dissolved in DMSO (vehicle; 5%, *v*/*v*) was applied intraperitoneally, and individual dehydrosilybins were applied orally for seven days at a dose of 50 mg/kg b.wt. After 24 h of the last application, the mice were sacrificed and blood from their *superior vena cava* was collected for further analyses. The livers were cleaned and stored as pieces in nitrogen until used. For RNA analysis, 100 mg of each tissue was immediately placed in 1.5 mL microfuge tubes containing RNAlater and stored following the manufacturer's protocol until the RNA isolation was performed.

All animal studies met the criteria for the care and use of animals in experiments and were approved by the Animal Research Committee of the 1st Faculty of Medicine, Charles University, Prague.

### 2.12. Statistical Analyses

All data are expressed as mean ± SEM. Depending on their normality, the data were analyzed either by the Student *t*-test or the Mann–Whitney rank sum test and Kruskal-Wallis ANOVA with Dunn's correction. Differences were considered statistically significant when *P* values were less than 0.05.

## 3. Results

### 3.1. The Effect of Silymarin Flavonoids and Related Compounds on HMOX and UGT1A1 Expressions and Activities in HepG2 Cells

In our *in vitro* screening studies, focused on evaluation of the possible modulating effect of silymarin flavonoids and related compounds on *HMOX1* and *UGT1A1* expressions, a wide array of compounds were used (Figure 1). Based on the MTT assays (Table 1), *HMOX1* and *UGT1A* mRNA expression analyses were performed with the nontoxic plus 1/2 of nontoxic concentrations for HepG2 cells.

**Figure 2:**
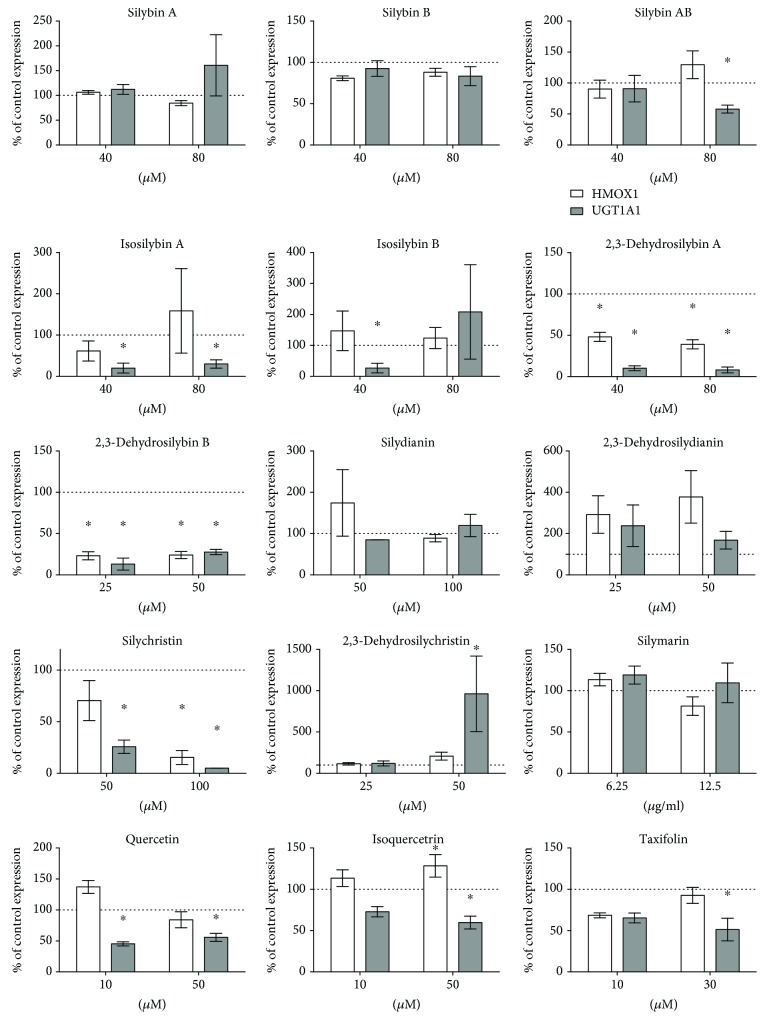
The effect of silymarin flavonoids and related compounds on *HMOX1* and *UGT1A1* expressions in HepG2 cells. mRNA expressions were analyzed after 4 h exposure of HepG2 cells to individual flavonolignans (in corresponding nontoxic concentrations). Data are expressed as percentage of control values. ^∗^ *P* < 0.05.

**Figure 3:**
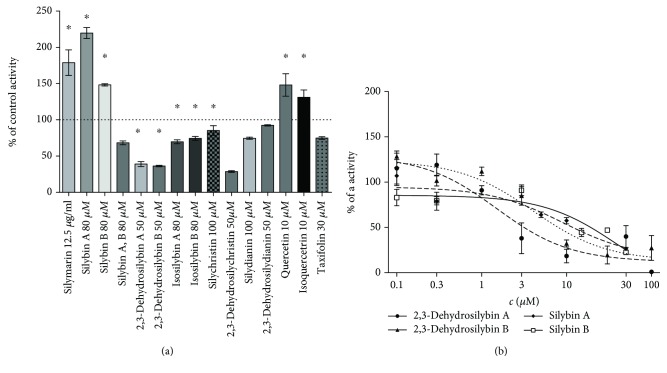
The effect of silymarin flavonoids and related compounds on (a) HMOX and (b) UGT1A1 activities in HepG2 cells. HMOX and UGT1A1 activities were analyzed after 24 h exposure of HepG2 cells to individual flavonolignans. HMOX activity was calculated as pmol CO/h/mg fresh weight and expressed as a percentage of the control values. UGT1A1 enzyme activity was based on relative light unit values and expressed as a percentage of the remaining activity. ^∗^ *P* < 0.05.

Significant *HMOX1* mRNA overexpression was observed for quercetin and isoquercitrin; on the other hand, *HMOX1* mRNA downregulation was present after exposure to 2,3-dehydrosilybins A and B (Figure 2). More importantly, the activity of HMOX was upregulated not only by quercetin and isoquercitrin but also after exposure to the silymarin complex *per se*, as well as silybins A and B. Downregulation of *HMOX1* mRNA expression by 2,3-dehydrosilybins A and B (Figure 2) was also reflected by decreased HMOX activity (Figure 3(a)).

Significant underexpression of *UGT1A1* mRNA was noted after exposure of HepG2 cells to the equimolar mixture of silybins A and B, to quercetin, isoquercitrin, taxifolin, and 2,3-dehydrosilybins A and B (Figure 2). These results were reflected by the inhibition of UGT1A1 activity, as determined for 2,3-dehydrosilybins A and B (IC_50_ values of 2.1 ± 0.2 and 4.1 ± 0.2 *μ*mol/L, respectively; Figure 3(b)).

### 3.2. The Effect of Silymarin Flavonoids and Related Compounds on Intracellular Concentrations of Bilirubin in HepG2 Cells

To investigate whether the effects of silymarin flavonoids and related compounds on HMOX and UGT1A1 are translated into phenotypic changes, we analyzed bilirubin concentrations within the HepG2 cells after 24 h exposure to individual compounds.

**Figure 4:**
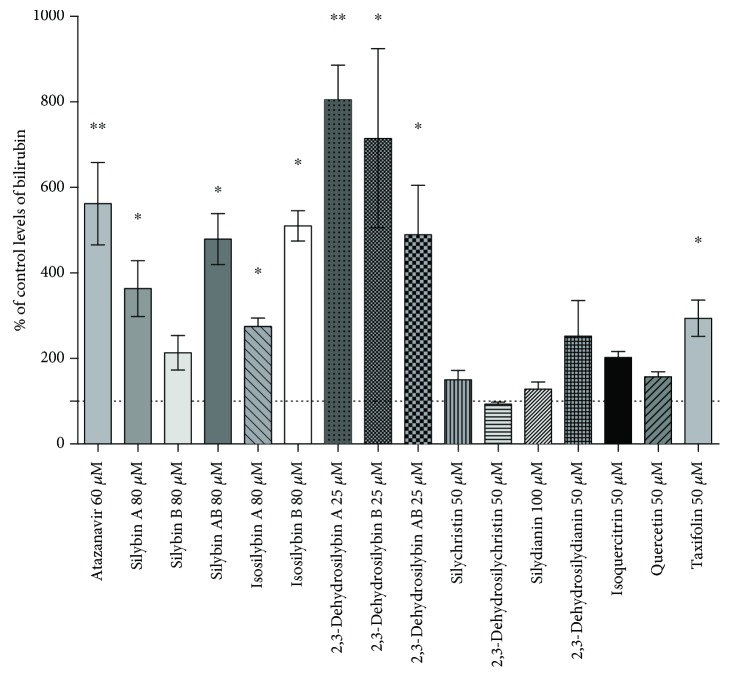
The effect of silymarin flavonoids and related compounds on intracellular concentrations of bilirubin in HepG2 cells. Control values are expressed as 100% and correspond to 2 pmol of bilirubin per mg of protein. ^∗^ *P* < 0.05; ^∗∗^ *P* < 0.005.

Most of silymarin flavonoids and related compounds significantly elevated intracellular bilirubin (Figure 4) in a dose-dependent manner (data not shown). Interestingly, the increase of intracellular bilirubin concentrations caused by exposure of both 2,3-dehydrosilybins A and B was even higher than that caused by atazanavir, a known inhibitor of UGT1A1 [40] (Figure 4).

Based on these *in vitro* results, 2,3-dehydrosilybins A and B were selected for the *in vivo* studies.

### 3.3. The Effect of 2,3-Dehydrosilybins A and B on Bilirubin Metabolism in Mice

#### 3.3.1. Concentrations of 2,3-Dehydrosilybins in Sera

**Figure 5:**
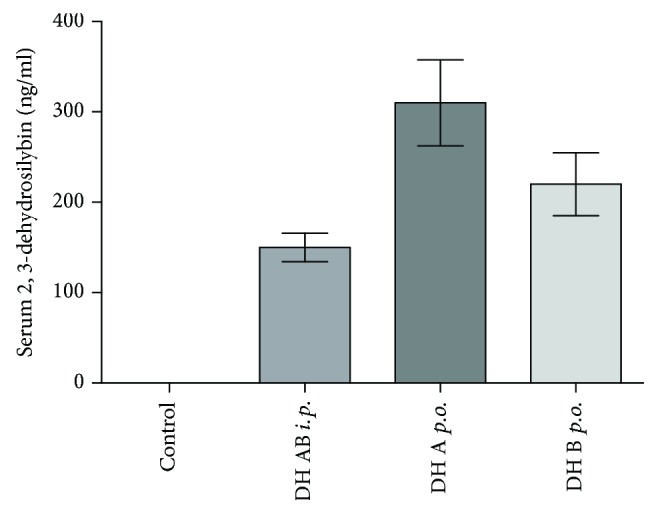
Serum concentrations of 2,3-dehydrosilybins after intraperitoneal and oral administration. 2,3-Dehydrosilybins (DH) were administered at a dose of 50 mg/kg b.wt. *i.p.*: intraperitoneal; *p.o.*: oral; b.wt.: body weight.

Since 2,3-dehydrosilybins have a low solubility in water and the poor oral bioavailability might contribute to the low clinical efficiency of silymarin in humans, we first measured concentrations of 2,3-dehydrosilybins in sera to verify whether they pass into the systemic circulation. Use of both application routes (*i.e.*, intraperitoneal as well as oral administration of 2,3-dehydrosilybins (50 mg/kg b.wt.)), after 24 h, resulted in a substantial appearance in the systemic blood, reaching serum concentrations up to 300 ng/mL (0.62 *μ*mol/L) (Figure 5).

#### 3.3.2. Hepatic HMOX1 and UGT1A1 mRNA Expressions

**Figure 6:**
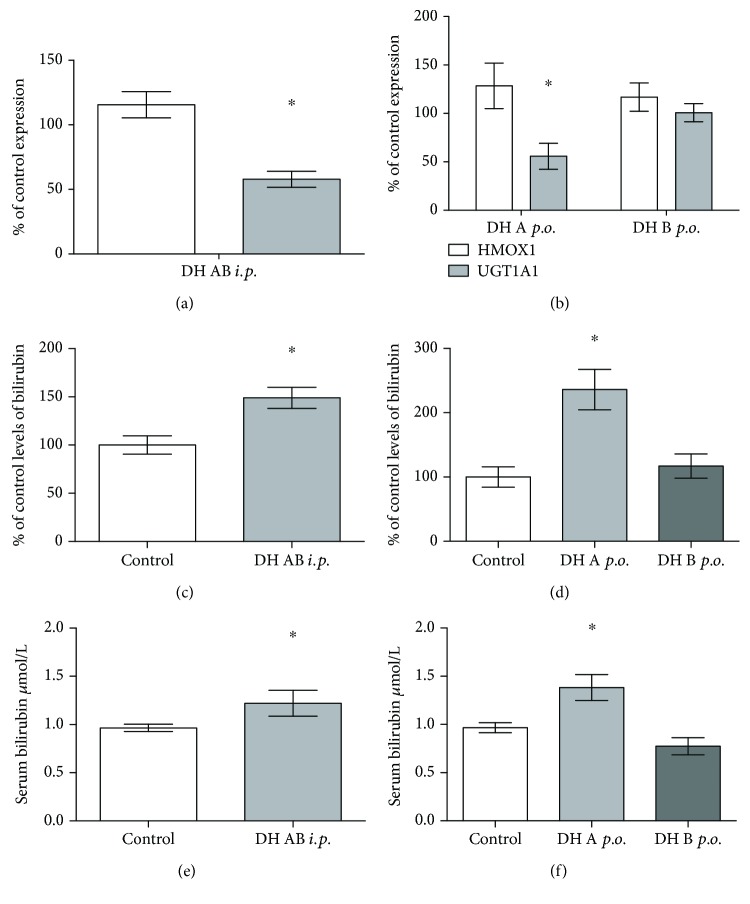
The effect of 2,3-dehydrosilybins on hepatic *HMOX1* and *UGT1A1* mRNA expressions and intrahepatic and systemic bilirubin concentrations. (a) *HMOX1* and *UGT1A1* mRNA expressions in mouse livers after intraperitoneal administration of a mixture of 2,3-dehydrosilybins A and B (50 mg/kg b.wt. for 7 days). (b) *HMOX1* and *UGT1A1* mRNA expressions in mouse livers after oral administration of either 2,3-dehydrosilybin A or B (50 mg/kg b.wt. for 7 days). (c) Intrahepatic bilirubin concentrations after intraperitoneal administration of a mixture of 2,3-dehydrosilybins A and B (50 mg/kg b.wt. for 7 days). (d) Intrahepatic bilirubin concentrations after oral administration of either 2,3-dehydrosilybin A or B (50 mg/kg b.wt. for 7 days). (e) Systemic bilirubin concentrations after intraperitoneal administration of a mixture of 2,3-dehydrosilybins A and B (50 mg/kg b.wt. for 7 days). (f) Systemic bilirubin concentrations after oral administration of either 2,3-dehydrosilybin A or B (50 mg/kg b.wt. for 7 days). ^∗^ *P* < 0.05. b.wt.: body weight.

Then we analyzed the effect of 2,3-dehydrosilybin administration on *HMOX1* and *UGT1A1* mRNA expressions in the livers of treated mice. Treatment with an equimolar mixture of 2,3-dehydrosilybins A and B (50 mg/kg b.wt.) administered intraperitoneally for 7 days led to a significant downregulation of *UGT1A1* mRNA expression in the liver (57 ± 19% of the controls) with no change in *HMOX1* expression (Figure 6(a)). Similar results were observed for oral treatment with 2,3-dehydrosilybin A (downregulation of *UGT1A1* mRNA expression to 55 ± 26% of the control, Figure 6(b)); while no effect was demonstrated for 2,3-dehydrosilybin B. None of the individual dehydrosilybins had any effect on *HMOX1* expression (Figure 6(b)).

#### 3.3.3. Intracellular and Systemic Bilirubin Concentrations

Intraperitoneal application of 2,3-dehydrosilybins A and B mixture significantly increased intracellular concentrations of bilirubin in the liver tissue (to 149 ± 24%, *p* < 0.05, Figure 6(c)). An even higher increase was observed after oral treatment of 2,3-dehydrosilybin A (to 236 ± 28%, *p* < 0.05; Figure 6(d)), whereas treatment with 2,3-dehydrosilybin B only led to a moderate but not significant elevation (Figure 6(d)).

Neither intraperitoneal nor oral administration of 2,3-dehydrosilybins caused any increase in markers of liver damage (ALT, AST, and ALP activities; data not shown).

Consistent with the results of intrahepatic bilirubin concentrations, intraperitoneal administration of the 2,3-dehydrosilybins A and B mixture also significantly elevated serum bilirubin concentrations (by 27%; 0.96 ± 0.09 vs. 1.22 ± 0.35 *μ*M; Figure 6(e)). Even a more pronounced elevation of serum bilirubin concentrations was observed after oral treatment with 2,3-dehydrosilybin A (by 44%; 0.96 ± 0.18 vs. 1.38 ± 0.29 *μ*M; Figure 6(f)). Treatment with 2,3-dehydrosilybin B did not cause any changes in serum bilirubin concentrations (Figure 6(f)).

#### 3.3.4. Hepatic Lipoperoxidation

**Figure 7:**
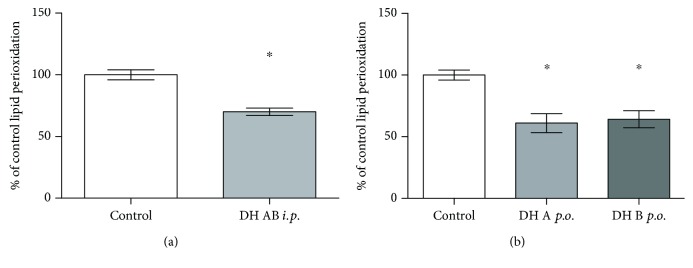
The effect of intraperitoneal and oral administration of 2,3-dehydrosilybins on lipoperoxidation in the liver tissue. (a) MDA concentrations in the mouse liver tissue after intraperitoneal administration of a mixture of 2,3-dehydrosilybins A and B (50 mg/kg b.wt. for 7 days). (b) MDA concentrations in the mouse liver tissue after oral administration of either 2,3-dehydrosilybin A or B (50 mg/kg b.wt. for 7 days). Data are expressed as a percentage of the control values. ^∗^ *P* < 0.05. b.wt.: body weight.

Treatment of mice with 2,3-dehydrosilybins had consistent inhibitory effects on lipid peroxidation in the liver tissue. MDA concentrations in the liver tissue were significantly reduced after intraperitoneal application of the mixture of 2,3-dehydrosilybins (to 70 ± 7% of the control; Figure 7(a)), and the same effect was also observed after oral administration of both 2,3-dehydrosilybins A and B (61 ± 15% and 64 ± 13% of the control, respectively; Figure 7(b)).

## 4. Discussion

Bilirubin, a bile pigment, for decades considered only an ominous sign of liver diseases, has been revisited as a potent antioxidant, immunosuppressive, and cytoprotective agent [2, 9, 10, 41]. Even single micromolar elevations of systemic concentrations of bilirubin, still within the physiological range, are associated with a substantial decreases of the risks of cardiovascular, cancer, and inflammatory diseases [42–44], and this association is clearly evident in subjects with Gilbert syndrome, characterized with mild systemic elevations of unconjugated bilirubin [9, 45]. This led to the suggestion to induce Gilbert syndrome iatrogenically in order to suppress the development of oxidative stress-related diseases [12].

As reported in our recent study, due to a dynamic equilibrium, a correlation between systemic and intracellular concentrations of bilirubin exists in the liver (and also probably in other organs and tissues) [37]. Thus, it is important to monitor intrahepatic concentrations of bilirubin when assessing potential hepatoprotective effects, in particular when considering the fact that hepatic UGT1A1 is the major enzyme affecting systemic concentrations of bilirubin [11]. Hepatic UGT1A1 is a biotransforming enzyme important not only for bilirubin but also for a variety of other endogenous substances as well as xenobiotics, including natural agents often used as nutraceuticals, including the flavonolignans and flavonols of the silymarin complex. In fact, inhibitory effects towards UGT1A1 were reported for silybin, a major flavonoid constituent of milk thistle extract [46]; indeed, the therapy with this substance has also been associated with marked unconjugated hyperbilirubinemia [19]. A significant increase in serum bilirubin levels was also reported in other human studies on patients with chronic hepatitis C receiving silybin therapy [20–23]. These clinical data are consistent with our findings, *i.e.*, increased intracellular as well as systemic concentrations of bilirubin upon exposure to silymarin flavonoids. It is important to emphasize that these effects differed substantially among individual flavonoids—dehydrosilybins A and B being the most efficient.

Despite generally low bioavailability of silymarin flavonoids, effective serum concentrations of both 2,3-dehydrosilybins A and B were detected in our study (up to 300 ng/mL (Figure 5), corresponding to concentrations of 0.62 *μ*mol/L). This value entirely fits to the mean peak plasma concentration of silybin, reached after oral administration of a 700 mg dose of silymarin (containing approximately 250 mg of silybin), which was 0.6 *μ*mol/L [46]. Very similar plasma silybin concentrations were reported in another human study (0.4, 1.4, and 4 *μ*mol/L after 360, 720, or 1440 mg of silybin administered daily for 7 days), with corresponding concentrations in the liver tissue [47], as well as in the studies by Barzaghi et al. (silybin plasma levels of 0.38 *μ*mol/L after administration of 240 mg of silybin given daily for 7 consecutive days) [48] and Wen et al. (plasma concentrations of 0.8 *μ*mol/L after oral administration of 600 mg of silymarin) [49].

In addition, silybin was demonstrated to potently inhibit UGT1A1, with the IC_50_ value correlating with the clinically relevant plasma concentrations [46]. Similar inhibitory concentrations were also reported by others for other silymarin flavonoids, although 2,3-dehydrosilybins were not tested in these studies [50]. These data are consistent with our findings, with the UGT1A1 IC_50_ value for 2,3-dehydrosilybin A = 2.1 *μ*mol/L and the mean serum concentration of 0.62 *μ*mol/L. Importantly, we found the UGT1A1 IC_50_ value for 2,3-dehydrosilybin A even lower than that reported for atazanavir (2.3 *μ*mol/L), the well-known hyperbilirubinemia-inducing drug [40] indicating the high bilirubinemia-enhancing potential of this flavonolignan.

Although the UGT1A1 IC_50_ values of 2,3-dehydrosilybin A are slightly higher than those reported for silybin, 2,3-dehydrosilybin A most efficiently increased both the hepatic intracellular and systemic concentrations of bilirubin. The reason for this might be that 2,3-dehydrosilybin A also affects other mechanisms implicated in hepatic bilirubin metabolism. Such an example may include the modulation of the basolateral bilirubin organic anion-transporting proteins OATP1B1/3, which are also inhibited by silymarin flavonoids [51].

2,3-Dehydrosilybins, together with other 2,3-dehydroflavonolignans, belong to the minor flavonoids of the silymarin complex, usually accounting for 1-2% of all flavonolignans (1.8% in the preparation used in the present work; Supplementary Figure 3). Their biological effects, however, might be of real clinical importance [28]. This is supported by a recent observation that 2,3-dehydrosilybins A/B significantly suppressed oxidative stress in *C. elegans* resulting in lifespan extension [32]. Although the effect on bilirubin metabolism was not investigated in this study, it is important to note that the antiaging effects of bilirubin have been demonstrated in another of our studies [7]. In this respect, it is also important to emphasize the antioxidant effects of 2,3-dehydrosilybins observed in our study. Indeed, decreased MDA concentrations in the livers of mice treated with 2,3-dehydrosilybins might have been mediated, at least partially, *via* increased intracellular bilirubin concentrations. It is thus likely that these antioxidant effects are not directly related to antioxidant activities of the flavonolignans per se, but rather parahormetic action is more important [52].

## 5. Conclusions

Natural silymarin flavonolignans contained in milk thistle and related flavonols, in particular 2,3-dehydrosilybins A and B, affect hepatic and serum bilirubin concentrations, as well as lipoperoxidation in the liver. This phenomenon might contribute to the hepatoprotective effects of silymarin observed in many, although not all clinical studies. Modulation of bilirubin metabolism by well-defined natural polyphenols can represent a safe chemopreventive approach against oxidative stress-mediated diseases including atherosclerosis, cancer, diabetes, or inflammatory diseases.

## Figures and Tables

**Table 1:** Inhibitory concentrations of individual silymarin flavonoids and related compounds.

Compound	IC_50_ (*μ*M)
Silybin A	>200
Silybin B	>200
Silybin AB	>200
Isosilybin A	148 ± 3
Isosilybin B	185 ± 4
2,3-Dehydrosilybin A	>200
2,3-Dehydrosilybin B	>200
Silychristin	>200
2,3-Dehydrosilychristin	>200
Silydianin	>200
2,3-Dehydrosilydianin	>200
Isoquercitrin	115 ± 4
Quercetin	105 ± 3
Taxifolin	>200

HepG2 cells were treated for 24 h with natural flavonoids in concentrations of 0.1-200 *μ*M. IC: inhibitory concentration, measured by the MTT test.

## Data Availability

The data used to support the findings of this study are available from the corresponding author upon request.

## Abbreviations

UGT1A1: UDP-glucuronosyl transferase
HMOX: Heme oxygenase
HPRT: Hypoxantin phosphoribosyl transferase
ALT: Alanine aminotransferase
AST: Aspartate aminotransferase
ALP: Alkaline phosphatase
MDA: Malondialdehyde
IC: Inhibitory concentration.

## Supplementary Materials

Supplementary Materials Supplementary Material: a detailed analytical HPLC-MS method for the determination of silymarin flavonolignans together with obtained data from the analyses, i.e., detailed composition of silymarin complex used in the study. Supplementary Table 1: primers used for gene expression studies.
