# Supplementary material for: Isolated Silymarin Flavonoids Increase Systemic and Hepatic Bilirubin Concentrations and Lower Lipoperoxidation in Mice
Source: Oxid Med Cell Longev. 2019 Feb 12;2019:6026902. doi: 10.1155/2019/6026902 (PMC6390243; doi:10.1155/2019/6026902)

**Isolated flavonoids of silymarin complex increase systemic and hepatic concentrations of bilirubin and lower lipoperoxidation in mice**

Šuk J^1^, Jašprová J^1^, Biedermann D^2^, Petrásková L^2^, Valentová K^2^, Křen V^2^, Muchová L^1^,

*Vítek L^1,3^

*^1^Institute of Medical Biochemistry and Laboratory Diagnostics, 1^st^ Faculty of Medicine, Charles University;*

*^2^Laboratory of Biotransformation, Institute of Microbiology of the Czech Academy of Sciences;*

*^3^4^th^ Department of Internal Medicine, 1^st^ Faculty of Medicine, Charles University;* (*all above Prague, Czech Republic*)

**HPLC and LC-MS analysis of silymarin**

*Experimental*

The content of taxifolin and flavonolignans in silymarin (Sigma-Aldrich), and the calibration curves of taxifolin and silybin were measured on a Chromolith RP-18e (100 × 3 mm) column (Merck), Chromolith RP-18e (5 × 4.6 mm) precolumn (Merck), mobile phase: 2% acetonitrile, 37% methanol, 61% water, 0.1% HCOOH; isocratic elution; flow rate 1.1 mL/min, 25°C. The content of taxifolin in silymarin was calculated from the calibration curve of taxifolin; the content of silybin A, silybin B, silychristin A, silychristin B + silydianin (co-eluting), isosilybin A, and isosilybin B were determined using the calibration curve of silybin (possible differences in the extinction coefficients of the individual flavonolignans were neglected).

The content of 2,3-dehydroflavonolignans in silymarin and standard solutions of 2,3-dehydrosilybin was determined using LC-MS measured on a Chromolith RP-18e (100 × 3 mm) column (Merck), Chromolith RP-18e (5 × 4.6 mm) precolumn (Merck), mobile phase: A = 5% acetonitrile, 0.1% HCOOH; B = 80% acetonitrile, 0.1% HCOOH; gradient: 0 min 20% B, 5 min 90% B, 6 min 90% B, 8-10 min 20% B; flow rate 0.4 mL/min, 25°C. The MS parameters were as follows: ESI interface voltage, 4.5 kV; detector voltage, 1.15 kV; nebulizing gas flow, 1.5 mL.min^-1^; drying gas flow, 15 mL.min^-1^; heat block temperature, 200°C; DL temperature, 250°C; negative scan mode 478.8-481.0 *m/z*; software LabSolutions ver. 5.75 SP2 (Shimadzu, Kyoto, Japan). The contents of 2,3-dehydrosilybin, 2,3-dehydrosilychristin and at least two additional 2,3-dehydroflavonolignans were determined using the calibration curve of 2,3-dehydrosilybin.

*Results*

Physical properties, that is to say, extinction coefficients and retention times of 2,3-dehydroflavonolignans differ substantially from that of flavonols and flavonolignans; therefore, two separate methods had to be used for proper silymarin analysis. While taxifolin together with the flavonolignans could be identified through comparisons with standards and quantified on HPLC (Fig. S1), the 2,3-dehydroflavonolignans were determined by LC-MS (Fig. S2). Besides 2,3-dehydrosilybin and 2,3-dehydrosilychristin, which were identified using standards, at least two additional 2,3-dehydroflavonolignans having the same molecular mass and UV-vis spectra were identified in the LC-MS chromatogram, but did not correspond to any of the standards available (2,3-dehydrosilydianin, 2,3-dehydroisosilybin). After quantification of all identified compounds, 17.5% of silymarin content in the measured sample remained unknown and probably accounts for polymeric flavonolignans. Altogether, the silymarin sample used in the present study contained 13.0% of silybin A, 17.9% silybin B, 14.7% silychristin A, 9.3% silychristin B + silydianin, 8.9% isosilybin A, 6.8% isosilybin B, 3.0% taxifolin, 1.9% 2,3-dehydrosilybin, 0.5% 2,3-dehydrosilychristin, as well as 6.5% of other non-identified 2,3-dehydroflavonolignans and 17.5% of other substances, probably polymers (Fig. S3).

**Suppl. Table 1. Primers used for gene expression studies.**

| ***HMOX 1*** |
| --- |
| 5´ GGGTGATAGAAGAGGCCAAGA-3´ |
| 5´ AGCTCCTGCAACTCCTCAAA-3´ |
| ***UGT1A1*** |
| 5´CAGAACTTTCTGTGCGACGTG-3´ |
| 5´GGGCCTAGGGTAATCCTTCAC-3´ |
| ***HPRT* (reference gene)** |
| 5´ CACTGGCAAAACAATGCAGAC 3 ´ |
| 5´ GGGTCCTTTTCACCAGCAAG 3 ´ |

*HMOX1*, heme oxygenase 1; *UGT1A1*, bilirubin UDP-glucuronosyl transferase 1A1; *HPRT*, hypoxantin phosphoribosyl transferase.

**Suppl. Fig. 1.** **HPLC chromatogram of flavonoids and flavonolignans in silymarin (Sigma) used.**


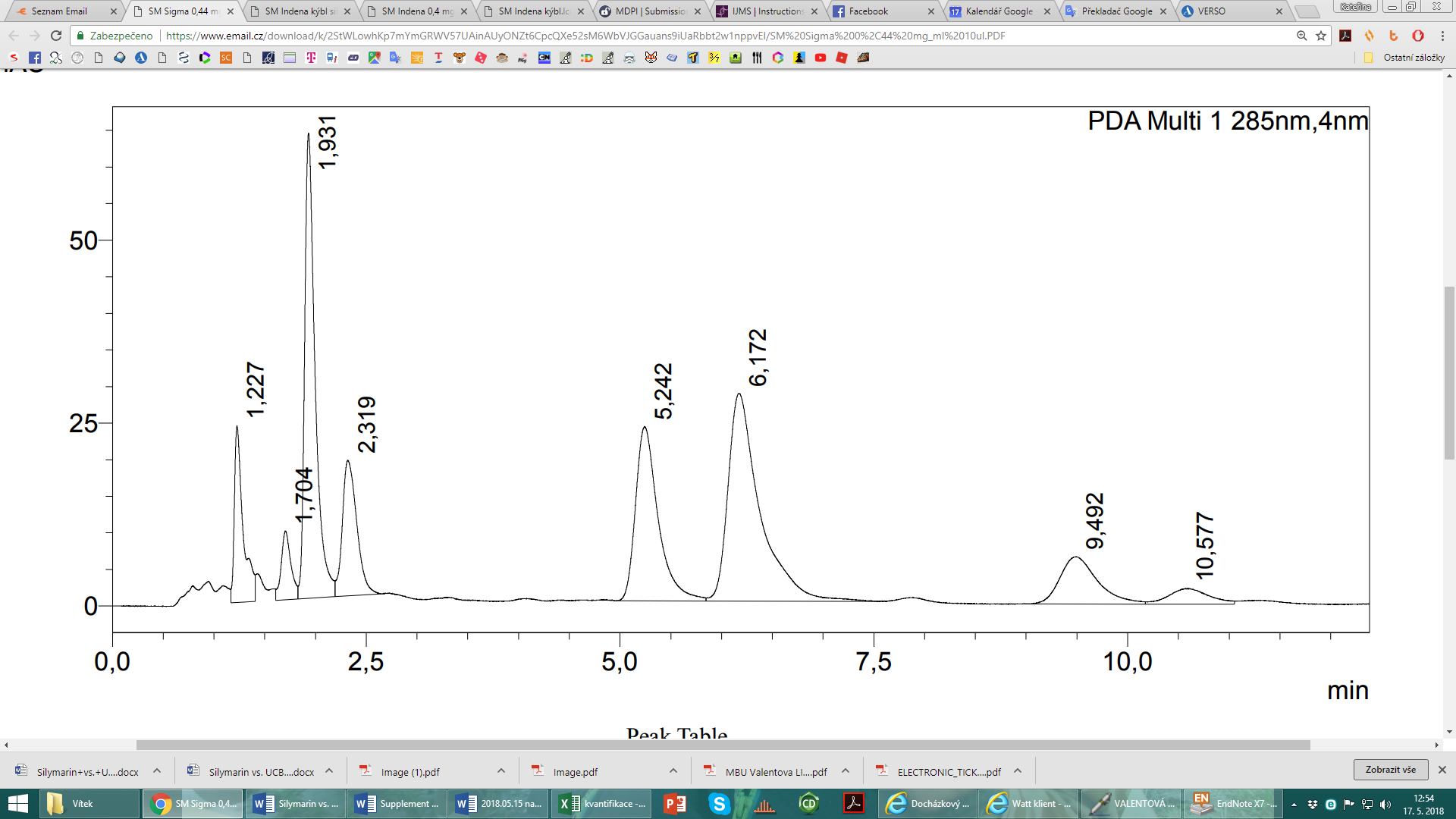


Taxifolin

Silychristin A

Silychristin B + silydianin

Silybin A

Silybin B

Isosilybin A

Isosilybin B

**Suppl. Fig. 2. LC-MS analysis of 2,3-dehydroflavonolignans in silymarin (Sigma) used in the study.**

**A**: UV-Vis detection at 360 nm, **B**: single ion monitoring at *m/z* 479 (negative mode), **C**. representative MS spectrum


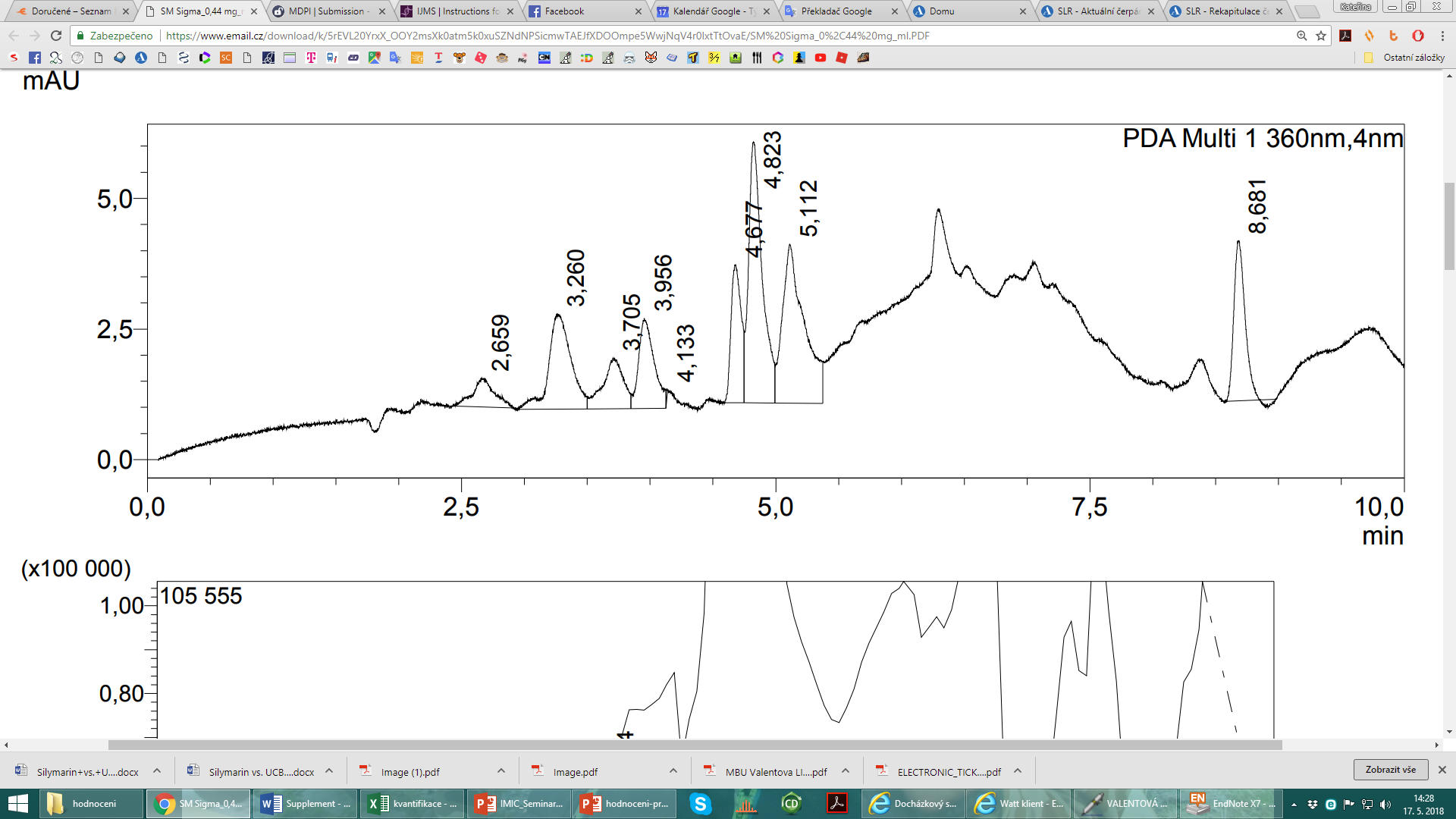


**B**

**A**


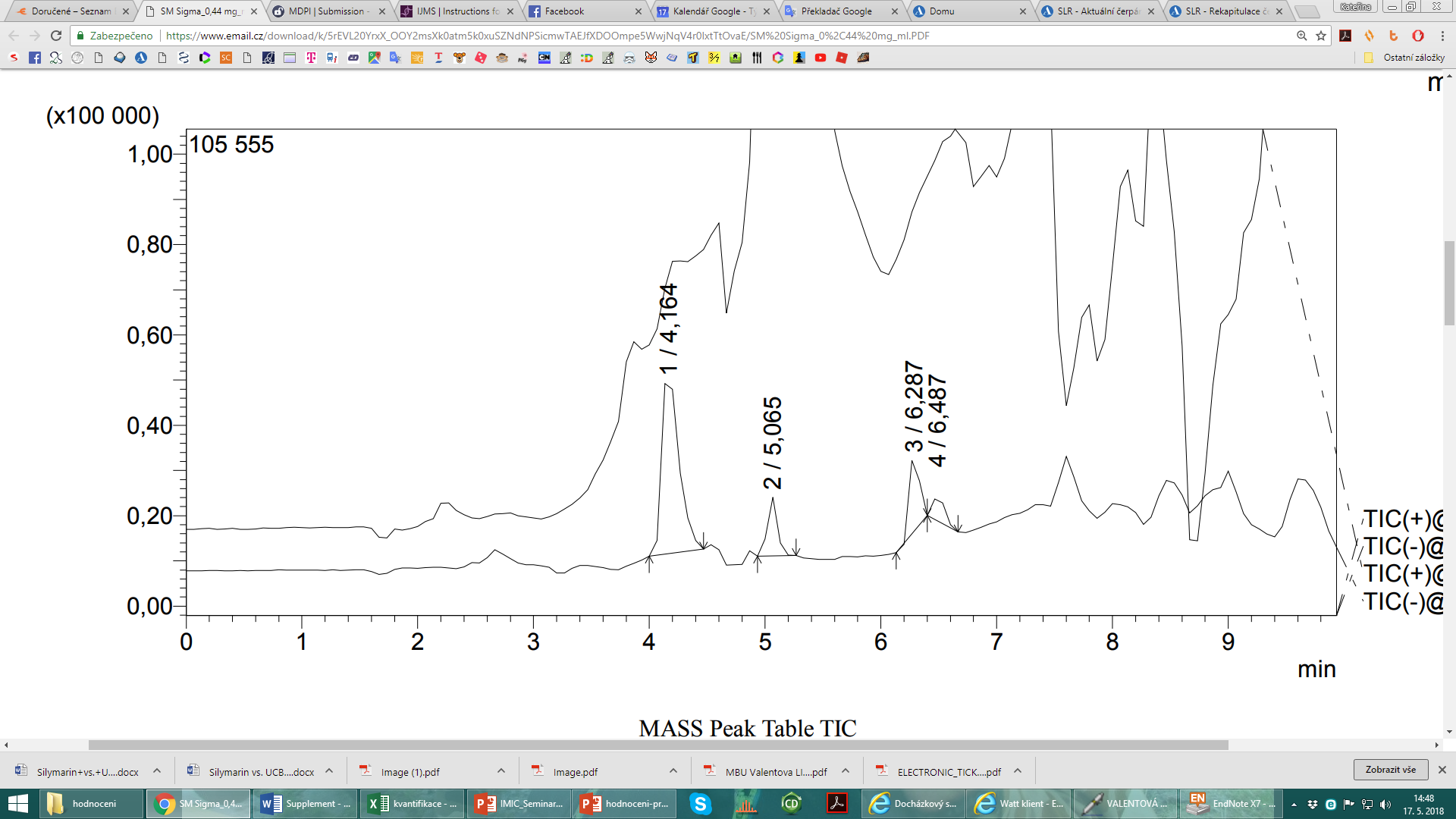


**C**

2,3-Dehydro-silychristin

2,3-Dehydro-silybin


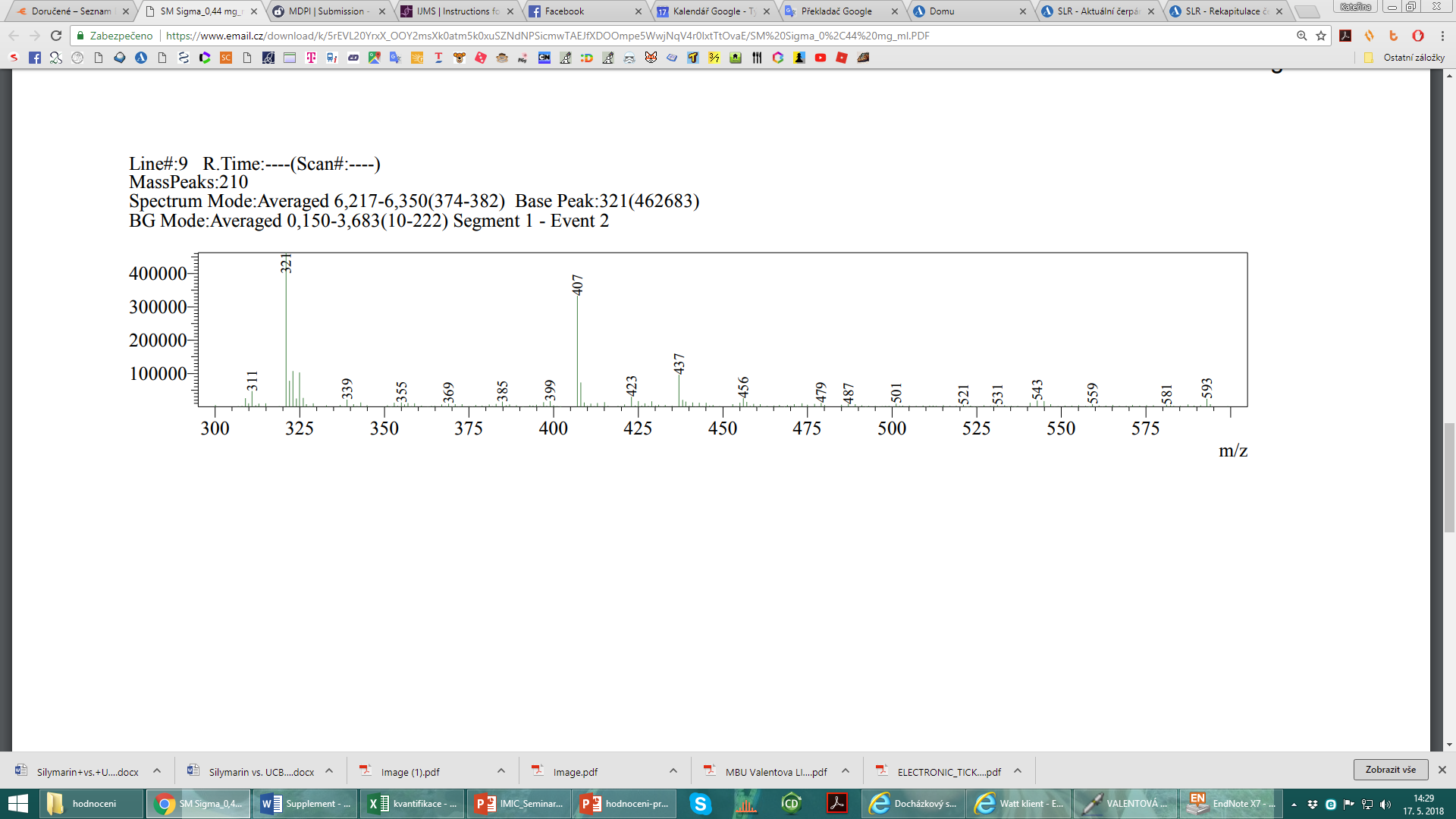


**Suppl. Fig. 3. Content of individual flavonoids in silymarin sample (Sigma) used in this study.**


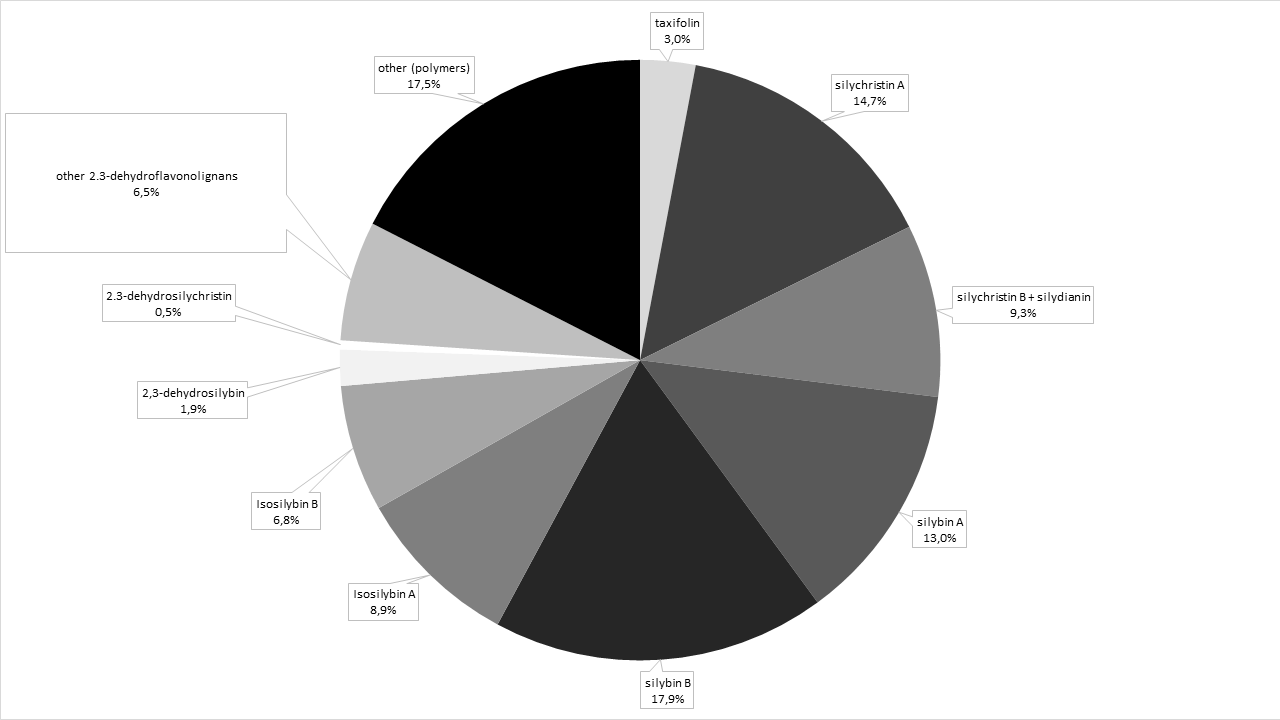

Supplement: Supplementary Materials — Supplementary Material: a detailed analytical HPLC-MS method for the determination of silymarin flavonolignans together with obtained data from the analyses, i.e., detailed composition of silymarin complex used in the study. Supplementary Table 1: primers used for gene expression studies. [file 6026902.f1.docx]
